# Impact of Balkan and Banat Donkey Milk on the Technological Process, Microbiological Quality, Composition, and Consumer Acceptability of Rolled Cheese

**DOI:** 10.3390/foods14122041

**Published:** 2025-06-10

**Authors:** Suzana Vidaković Knežević, Jelena Vranešević, Nenad Popov, Slobodan Knežević, Dragana Ljubojević Pelić, Milica Živkov Baloš

**Affiliations:** Scientific Veterinary Institute “Novi Sad”, 21000 Novi Sad, Serbia; jelenababic@niv.ns.ac.rs (J.V.); nenad.p@niv.ns.ac.rs (N.P.); slobodan.knezevic@niv.ns.ac.rs (S.K.); milica@niv.ns.ac.rs (M.Ž.B.)

**Keywords:** alternative milk sources, traditional/artisanal cheese, autochthonous donkey breeds

## Abstract

Donkey milk is well known for its beneficial properties for human health, making it a valuable ingredient in the production of value-added cheese. Rolled cheese, a type of pasta filata cheese, is traditionally produced in the northern part of Serbia. In this study, we produced rolled cheese by adding a certain amount of donkey’s milk from the Balkan and Banat breeds to cow’s milk. The rolled cheese samples were analyzed for their microbiological quality, chemical composition, content of essential and trace elements, as well as sensory characteristics. Adding 10% and 20% donkey’s milk had no effect on the microbiological quality or hedonic scale of rolled cheeses compared with rolled cheese made from raw cow’s milk. However, the addition of donkey’s milk partially affected the chemical composition and mineral profile of the cheeses. The fat, fat in dry matter, calcium contents, and the ratio of calcium and phosphorus significantly (*p* < 0.05) decreased with the addition of donkey’s milk, while the ash, salt, sodium, and potassium contents significantly (*p* < 0.05) increased. The assessors successfully distinguished the rolled cheeses containing donkey’s milk from those made with cow’s milk, encouraging the future production of value-added cheese.

## 1. Introduction

Donkey milk is a food product expected to see increasing demand in the future, considering the rising prevalence of allergic reactions to cow’s milk [1], as well as consumers’ growing interest in incorporating new types of food into their diets. What distinguishes donkey milk is its notable similarity to human milk [2,3,4], making it a promising alternative for the nutrition of infants and children with cow’s milk protein allergies [5,6]. It is a good source of proteins (range 1.22–2.14%), amino acids, fatty acids, minerals, and trace elements [4,7,8]. Furthermore, it has a favorable mineral profile and is particularly rich in calcium (Ca; range 466.68–947 mg/L), phosphorus (P; range 369.0–589.0 mg/L), sodium (Na; 157.0–910.55 mg/L), potassium (K; range 405–2009.67 mg/L), magnesium (Mg; range 54.0–248.88 mg/L), zinc (Zn; average 2.36 mg/L), and copper (Cu; average 0.027 mg/L) [2,4,9,10], which may enhance the nutritional value of dairy products made from it. Additionally, donkey milk is characterized by a high level of lactose, low-fat content (range 0.16–1.20%), and is rich in bioactive compounds including lysozyme, lactoferrin, lactoperoxidase, and immunoglobulins [4,11,12], which contribute to its antimicrobial and immunomodulatory benefits, making it a promising ingredient in the production of high-quality cheese. Previous studies on the microbiological quality of donkey milk indicate a wide range in total bacterial count (7 to 90,000 CFU/mL) [13,14] but have generally shown an absence of pathogenic microorganisms [13], likely due to the milk’s antimicrobial properties. Nonetheless, occasional findings raise hygiene concerns, necessitating careful handling [15]. From a sensory standpoint, the low-fat content of donkey milk may affect the flavor and texture of cheese made from it [16]. Moreover, its low casein concentration (total casein content in donkey milk is in the range of 6.6–10.3 g/L, while in cow and goat milk, the average content is 27.2 and 25.0 g/L, respectively) poses a challenge for cheese production, primarily due to coagulation difficulties [17,18]. Despite these limitations, recent studies have demonstrated that cheese can be successfully produced from donkey milk through various technological interventions, such as using specific coagulants [19,20,21], optimizing coagulation conditions [20], blending with milk from other species [20,22,23], or enhancing protein cross-linking with enzymes like transglutaminase [14,24].

Faccia et al. [20] managed to produce acceptable cheese using calf rennet and modified vat operations, while Cosentino et al. [25] observed that the addition of 5% donkey milk influenced sensory characteristics in cow milk Caciotta cheese after ripening. These studies highlight that although donkey milk is not a direct competitor to cow’s milk, it holds promise as a functional ingredient with added value and niche market potential.

The Balkan donkey, an indigenous breed in Serbia, has been studied over the last decade in research examining the microbiological quality of its milk, its antimicrobial potential, and the characteristics of extra-hard cheese produced by mixing this milk with goat’s milk in a 60:40 ratio [23,26,27,28]. More recently, the Banat donkey breed, which is more robust and productive than the Balkan donkey, has also been included in research [29]. These breeds are well adapted to local environmental conditions and are valued for their resilience, particularly for their milk. Both breeds have been studied regarding the nutritional composition of their milk and the presence of essential minerals, trace elements, and potentially toxic elements [10,30]. To date, no research has been conducted comparing the quality and characteristics of milk from these two breeds, primarily due to the difficulty of collecting separate milk samples, as the breeds are kept together in the reserve. This will certainly represent a challenge for future studies. Incorporating milk from these breeds into traditional cheese production could contribute to the preservation and promotion of local genetic resources, as well as improve the microbiological, nutritional, and sensory qualities of the resulting cheeses.

Cheese production is one of the oldest and most widespread methods of milk preservation, characterized by a significant diversity of regional, cultural, and technological variations [31,32]. Among countless cheese varieties, “rolled cheese” is a specific type of traditionally produced cheese from the Vojvodina region (northern part of Serbia), known for its unique texture and other attributes often determined by the type of milk used in its production. It belongs to the group of fresh cheeses made from stretched curd (pasta filata) [33]. Commonly used milk types in the production of rolled cheese include cow’s, sheep’s, and goat’s milk, or mixtures of these in various proportions. The production of this cheese involves manually stretching the elastic curd mass obtained after spontaneous fermentation of raw milk, to which a small amount of enzyme is added for coagulation once a certain level of acidity is reached. The stretched curd is then salted and formed into the characteristic rolls by which it is recognized. The use of donkey milk from indigenous breeds holds significant scientific and commercial potential, considering the specific properties of this type of milk. Although research has been conducted on the chemical composition and mineral profile, technological procedures, microbiological characteristics, sensory acceptability, and sustainability of rolled cheese from cow’s milk [34,35,36], to our knowledge, no research has yet been carried out on the addition of donkey milk in the production of this type of cheese. With the increasing market demand for new and innovative products, such research is of great importance.

The aim of this study is to assess the acceptability of rolled cheese by sensory panel participants when produced with varying proportions of added donkey milk from the Balkan and Banat breeds, with a special focus on the technological production process. Additionally, the microbiological quality, chemical composition, essential and trace elements content, and sensory characteristics of the resulting cheese were examined. The results will provide insights into the feasibility of using donkey milk in the production of innovative products, which may contribute to the promotion of artisanal cheese-making and rural economic development.

## 2. Materials and Methods

### 2.1. Milk Collection

Raw donkey milk was collected in December 2024 from Banat and Balkan donkeys raised extensively at the Special Natural Reserve Zasavica, located in Vojvodina, the northern region of Serbia, following the procedure described in Ljubojević Pelić et al. [10]. Donkey milk samples from Banat and Balkan donkey breeds were combined to create a composite sample, reflecting the overall milk quality of the herd, as the two breeds are raised together within the reserve. Cow’s milk was collected from Holstein Friesian cows raised on a farm located in Vojvodina, after mechanical milking. Until cheese making, the milk was kept at 4 °C.

### 2.2. Cheese Making

The rolled cheeses were produced at a small dairy plant in Bačka, one of the districts of the autonomous province of Vojvodina, Serbia. The physico-chemical composition, pH, essential minerals, and trace elements in cow’s and donkey’s milk used for cheese making are summarized in Table 1.

The total volume of milk used to make rolled cheeses was 10 L per treatment. The rolled cheese obtained using only cow’s milk served as the control, while the experimental rolled cheeses were produced with the addition of 10% and 20% donkey’s milk. Initially, the temperature of raw milk was heated to 40 °C. Powder rennet (0.3 g; Caglificio Clerici, Como, Italy), dissolved in a small amount of water, was then added to the milk. The milk was left to form curds for 60 min (100% cow’s milk), 260 min (90% cow’s milk and 10% donkey’s milk), and 310 min (80% cow’s milk and 20% donkey’s milk). The notably extended coagulation times observed in curds with increasing proportions of donkey’s milk are likely due to the compositional characteristics of donkey milk. The formed curd was cut into cubes (approximately 2 cm^3^) and left for another 40 min. The whey was gently removed, and the curd was additionally cut and mixed with the remaining whey. Once again, the curd was left for approximately 30 min until the whey became white. Using a cotton cloth, the remaining whey was drained, after which the curd was stretched in hot water at 80 °C using a wooden spoon. The hot curd was hand-molded into a thin (5 mm) sheet. The sheet was hand-salted and hand-folded to form a rectangular form, which was then rolled from the sides to form two rolls. The rolls of cheese were wrapped into cotton cloth and left to drain for 24 h at 4 °C. The rolled cheeses were removed from cotton cloth, wrapped into cling film, and stored at 4 °C without undergoing ripening. All analyses were performed within 24 h.

### 2.3. Microbiological Analysis

A total of 25 g of rolled cheese samples were homogenized using a Stomacher (Mayo International SRL, Novate Milanese, Italy) for 2 min in 90 mL of sterilized peptone water (Biokar Diagnostics, Beauvais, France). Decimal dilutions were prepared, followed by enumeration of total mesophilic count (TMC) on plate count agar (PCA, CM0325, Oxoid, Basingstoke, UK), incubated at 30 °C for 72 h [37]; *Enterobacteriaceae* on violet red bile glucose agar (VRBGA, CM1082, Oxoid, UK), incubated at 37 °C for 24 h [38]; *Escherichia coli* β-glucuronidase positive on tryptone bile glucuronide agar (TBX, CM0945, Oxoid, UK), incubated at 44 °C for 24 h [39]; and coagulase-positive staphylococci on Baird Parker agar (Biokar Diagnostics, Beauvais, France), incubated at 37 °C for 24–48 h [40]. Detection of foodborne pathogens, *Salmonella* spp. and *Listeria monocytogenes*, was carried out following ISO 6579-1 [41] and ISO 11290-1 [42], respectively.

### 2.4. Chemical Analysis

#### 2.4.1. Physico-Chemical Analysis

The total solids content was determined by oven drying in a laboratory oven at 102 °C, according to the ISO method [43,44]. Fat content was measured using Gerber’s volumetric method [45]. Fat content in dry matter was calculated based on the moisture and fat content results. Total nitrogen content was determined using the Dumas method on Rapid Exceed Analyzer (Elementar, Langenselbold, Germany). Protein content was calculated using a factor of 6.38 [46]. The pH was measured at 20 ± 0.5 °C using an electronic pH meter (Consort C 830, Turnhout, Belgium) calibrated with standard buffer solutions. The ash content was determined by dry ashing the samples in a muffle furnace at 550 °C, according to the method of A.O.A.C. [47,48]. Sodium chloride (salt) was calculated as sodium content × 2.5 (%).

#### 2.4.2. Essential Minerals and Trace Elements Analysis

Sample Preparation: Milk and cheese samples were prepared for ICP-MS analysis using the acid digestion method in the Ethos system, Microwave Labstation (Milestone s.r.l., Sorisole, Italy). In brief, 1 g of the milk and cheese sample was mixed with 8 mL of diluted HNO_3_ acid (2 parts of 65% *w*/*w* acid and 1 part of deionized water) and 2 mL of H_2_O_2_ (30% *w*/*w*). The mixture was then digested at 180 °C and 40 bars for 30 min. After digestion, the resulting clear acid solutions were quantitatively transferred to volumetric flasks and diluted with deionized water to a final volume of 25 mL. The blank sample was prepared similarly, excluding the cheese sample. All samples were prepared in duplicate.

Equipment and Analysis—Analysis of Mineral Elements: The elements of interest were determined using Agilent ICP-MS 7700 (Agilent Technologies, Santa Clara, CA, USA), equipped with collision/reaction cell technology (Octopole Reaction System, ORS) to minimize spectral interferences. Single-element standards of Ge, Rh, Lu, and Ir (all from CPI International, Amsterdam, The Netherlands) were used to prepare the internal standards. Calibration standards were prepared using single-element standards of Ca, P, Na, K, Mg, Fe, Zn, and Cu (all from AccuTrace™ Reference Standards, New Haven, CT, USA). All standards were prepared daily.

### 2.5. Sensory Evaluation of Rolled Cheeses

Female and male staff members of the Scientific Veterinary Institute “Novi Sad”, aged between 20 and 60 years and consumed cheese regularly, were recruited to evaluate rolled cheeses as members of the consumer panel. Before sensory analysis, the assessors completed a questionnaire that included information about sex, age, and familiarity with donkey milk products. A short training session was conducted to familiarize consumers with the descriptors.

**Figure 1:**
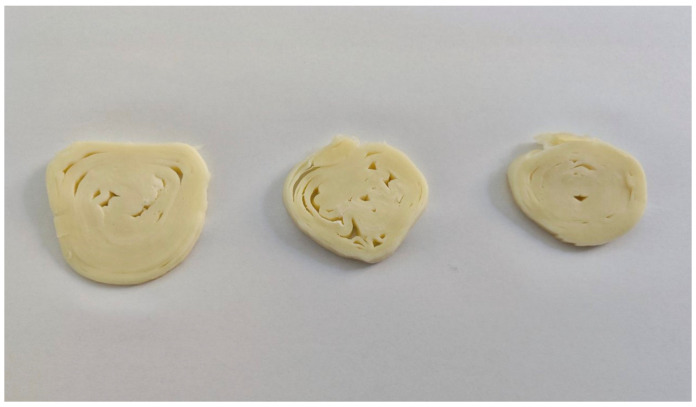
Examples of rolled cheese with cow’s milk (**left**), with 10% donkey’s milk (**middle**), and with 20% donkey’s milk (**right**), sliced for sensory analysis.

The cheese samples were removed from the refrigerator and cut into slices (Figure 1). All samples were assigned with three-digit random numbers on white plastic plates and served to consumers in individual booths at room temperature.

In the evaluation questionnaire, consumers were asked to evaluate color, texture, aroma, taste, and overall liking using a five-point hedonic scale (1 = dislike extremely and 5 = like extremely).

A triangle test was conducted to evaluate the rolled cheeses made with the addition of donkey milk. The panelists were asked to identify the different rolled cheeses. Rolled cheese samples containing a certain amount of donkey milk (10% and 20%) were presented alongside rolled cheese samples made of 100% cow’s milk as the reference sample.

To refresh the participants’ palates, both water and unsalted crackers were provided.

### 2.6. Statistical Analysis

All measurements were performed in duplicate, and the results were expressed as mean value ± standard deviation. To analyze variations of the results, ANOVA was including F-test was used. Analysis was performed using the software package Microsoft Office Excel 2007. Differences between the means at a probability level of *p* < 0.05 were considered statistically significant.

The expanded statistical table of Roessler et al. [49] was used to determine the significance of differences between rolled cheeses obtained from the triangle test.

## 3. Results and Discussion

### 3.1. Microbiological Status of Rolled Cheese

Although milk from healthy animals is considered sterile inside the udder, contamination with microorganisms during milking is unavoidable. Sources of different microorganisms include air, soil, feed, water, teats, worker’s hands, milking, and storage equipment [50,51,52]. However, initial contamination of raw milk can be kept low through proper animal hygiene, environmental and milking hygiene, and proper storage of milk [50,52,53].

Raw milk may be an ideal medium for microbial growth and may contain various microorganisms, including spoilage and pathogenic bacteria [54]. Therefore, raw milk cheeses have been considered microbiologically unsafe due to the absence of thermal treatment in the process of cheese making [55]. In addition, they may pose a potential risk and enable the survival and growth of microorganisms, including pathogens, due to their relatively high moisture content, pH values that allow pathogen survival and growth, and low salt concentrations. During production, the curd processing temperature was characteristic of the type of cheese and was around 40 °C, which created conditions for the survival of pathogenic bacteria. The use of commercial cultures enables a rapid decrease in pH value, thereby slowing down or stopping the growth of pathogens. It is also important to note that during the production of pasta filata cheeses, the heat treatment of the curd in hot water can have a pasteurization effect on the final product and reduce the growth of undesirable microorganisms [56], which was also the case in our study, as a temperature of 80 °C was used.

The microbiological status of rolled cheeses is summarized in Table 2. The populations of mesophilic bacteria did not show a significant difference among rolled cheeses (*p* = 0.717). This suggests that the addition of donkey milk does not significantly influence the overall microbial load of mesophilic organisms during the cheese-making process. This may be attributed to similar microbial loads in the raw milk and similar processing conditions in all groups. The mesophilic bacteria in rolled cheeses may have derived from raw milk, equipment, accessories, and workers’ hands being in contact with cheese during the cheese-making process. Our previous study showed that the donkey milk from the Special Natural Reserve Zasavica, stored at 4 °C after hand milking, contained mesophilic bacteria up to 5.34 ± 0.26 log_10_ CFU/mL [57]. Additionally, the mesophilic bacterial population in raw milk cheeses is dominated by lactic acid bacteria (*Lactobacillus*, *Lactococcus, Leuconostoc*, and *Enterococcus*) and non-starter lactic bacteria. The non-starter lactic acid bacteria can survive cleaning procedures in the form of biofilms in the dairy plant environment and be a potential source of cheese contamination [58].

The populations of *Enterobacteriaceae* did not show significant differences among rolled cheeses (*p* = 0.291). This suggests that the inclusion of donkey milk, known for its antimicrobial properties due to compounds such as lysozyme and lactoferrin [59], did not lead to a significant reduction in these microorganisms during production. Contrary, in the mentioned study, a significant reduction in the number of microorganisms was achieved when extracted lysozyme and lactoferrin were used in cheese production. *Enterobacteriaceae* species are recognized as indicators of milking and cheese-making hygiene. Some members of this bacterial family are potential pathogens and can cause severe foodborne outbreaks [60], while others can adversely affect organoleptic features and cause blowing due to their ability to catabolize proteins and lipids and produce gas [32,60,61].

One gas gas-producing strain is *Escherichia coli* [61], whose population slightly increased with the addition of donkey’s milk, but with no significant difference among rolled cheeses (*p* = 0.140). Belonging to the *Enterobacteriaceae* family, *Escherichia coli* is also used as an indicator of fecal contamination in food [61] and has previously been responsible for foodborne outbreaks linked with the consumption of raw milk cheese [62,63].

Although coagulase-positive staphylococci are oftentimes detected in raw milk cheeses and have been a food safety problem, with the prevalence ranging from 23.53% [64] to 87.32% [65], the number of coagulase-positive staphylococci was below the limit of quantification (<1.00 log_10_ CFU/g) in all rolled cheese samples, indicating good production hygiene.

Furthermore, the pathogens *Salmonella* spp. and *Listeria monocytogenes* were not detected in any of the rolled cheese samples. This could be due to good production hygiene or the well-known antibacterial properties of donkey milk [66,67,68].

All rolled cheese samples complied with the food safety and process hygiene criteria of Commission Regulation (EC) No 2073/2005 [69]. However, despite the abovementioned antimicrobial components of donkey milk, its addition at 10% and 20% did not significantly influence the microbiological safety or quality of the produced cheeses in our study. It is possible that the concentrations used were insufficient to produce a significant antimicrobial effect, or that the short production and ripening periods did not allow for these effects to be manifested.

### 3.2. Chemical Composition and Mineral Profile of Rolled Cheeses

The compositions of rolled cheeses are given in Table 3, while the content of essential minerals and trace elements are presented in Table 4. By analyzing the variance of the obtained results, it can be concluded that the values between the groups are defined by the type of rolled cheeses. The results did not show statistically significant differences for the following parameters: moisture content, protein content, protein content in dry matter, pH values, and the contents of P, K, Mg, Zn, Cu, and Fe. The obtained values for the F-test were lower than the F-critical ones, and the *p* values were higher than 0.05 (Table 3 and Table 4). Statistically significant differences were observed in the measured values of fat content, fat in dry matter, ash, salt, Ca, Na, and the Ca/P ratio between the defined types of rolled cheeses. The F-test values were significantly higher than the F-critical ones, and *the p*-values were lower than 0.05.

The chemical composition of the produced cheeses is presented in Table 3. The addition of donkey milk partially influenced the chemical composition of the cheese, which is consistent with previous research [21,23]. It is known that the chemical composition of donkey milk can vary depending on breed, diet, age, number of lactations, season, farming system, climatic conditions, and the animal’s health status [4,30,70,71]. Previous studies have shown that, compared to ruminant milk, donkey milk contains lower levels of dry matter, ash, fat, and protein [4,7,30,72]. The moisture content increased with the inclusion of donkey milk, although the differences were not statistically significant (*p* = 0.125). Donkey milk naturally has a higher water content compared to cow milk, which explains this observation. This increase in moisture may contribute to a softer and more elastic cheese matrix [73]. Our previous study [30] showed that the dry matter content of donkey milk from Zasavica ranged from 7.20% to 9.52%, which aligns with earlier findings [2,3,7,67,74,75,76,77,78,79,80,81,82]. The total fat content showed a statistically significant decrease (*p* < 0.05) with the inclusion of donkey milk, which is expected due to its above-mentioned naturally lower fat content. Additionally, the fat-in-dry-matter values followed a similar trend across all rolled cheese samples (*p* < 0.05). This is particularly relevant for labeling and product classification, especially in countries such as Serbia where cheese categories are regulated based on fat content in dry matter [83]. According to the obtained results, cheese made solely from cow milk falls into the full-fat category, while cheeses containing donkey milk are classified as semi-fat cheeses. The fat content in donkey milk from the Balkan and Banat breeds ranged between 0.10% and 1.00% [30], confirming that low-fat content is a characteristic attribute of donkey milk [84,85]. The protein content did not differ significantly among the cheese groups (*p* = 0.186), although a slight increase in protein content was observed with a higher proportion of donkey milk in the cheese. A similar trend was noted for protein in dry matter (*p* = 0.168). This result is somewhat unexpected, considering the lower protein content in donkey milk compared to cow milk [30,85]. In donkey milk from the Banat and Balkan breeds, protein content ranged from 1.17% to 2.07% [30]. The ash content, which represents the total mineral residue, was significantly affected by the addition of donkey milk, although the results were unexpected. Specifically, the highest ash content was observed in the cheese containing 10% donkey milk, followed by cheese with 20% donkey milk, and the lowest ash content was found in cheese made exclusively from cow milk. Salt content followed a similar pattern and also showed statistically significant differences between groups (*p* < 0.05). This outcome may be attributed to the fact that salting was performed manually during cheese production. Previous research [2,3,7,67,74,76,77,78,79,81,82] indicates that ash content in donkey milk ranges from 0.28% to 0.51%, which is approximately half the value typically found in cow milk. This lower mineral content is a beneficial attribute of donkey milk, as it may reduce renal load [85]. The pH of the produced rolled cheeses did not differ significantly between the examined samples (*p* = 0.448), although a slightly higher pH was observed in cheeses containing donkey milk. Donkey milk is known to have a naturally higher pH, ranging from 6.82 to 7.46 [2,3,7,30,74,79,80,82], compared to cow milk, which likely contributed to this effect. The nearly neutral pH of donkey milk is associated with its lower casein and phosphate content [8]. This can influence the sensory characteristics and shelf life of cheeses made with donkey milk [16,86].

The mineral composition of the produced rolled cheeses is presented in Table 4. Minerals are essential micronutrients that play vital roles in various biological processes, human metabolism, bone development, and enzymatic activity [87], making their presence in dairy products of considerable nutritional importance. Donkey milk, recognized for its distinctive composition and health-promoting properties, exhibits a favorable mineral profile [10] that can enhance the nutritional quality of dairy-derived products. Variations in mineral content are influenced by several factors, including breed, diet, and environmental conditions [4,88], emphasizing the importance of evaluating milk from different donkey breeds, such as those native to the Balkan and Banat regions. When incorporated into processed cheese, these mineral characteristics of donkey milk can influence not only the nutritional value but also the sensory attributes of the final product [23,89]. Therefore, assessing the mineral composition of rolled cheese enriched with Balkan and Banat donkey milk is critical for determining its potential as a functional food with added health benefits.

The highest calcium concentration was found in the control cheese made from 100% cow milk, with a significant decrease observed in cheeses containing donkey milk (*p* < 0.05). This result aligns with previous studies showing that cow milk generally contains higher levels of calcium than donkey milk [10]. A similar trend was observed for phosphorus, although the differences were not statistically significant (*p* = 0.187). As a result, the Ca/P ratio decreased in cheeses with added donkey milk (*p* < 0.05). Maintaining a balanced calcium-to-phosphorus ratio is essential for optimal mineral absorption and bioavailability [90,91,92]. Sodium content varied significantly among the groups (*p* < 0.05), likely due to the manual salting process used during cheese production. In contrast, potassium levels were significantly higher (*p* < 0.05) in cheeses containing donkey milk, which contributed to the improvement of the Na/K ratio, a key indicator of cardiovascular health [93]. A more favorable Na/K ratio enhances the nutritional value of the cheese and supports its potential role in heart-healthy diets. Magnesium content did not differ significantly among the tested cheese groups (*p* = 0.018). Additionally, zinc and copper levels showed minimal variation across all samples, with *p*-values of 0.181 and 0.246, respectively, suggesting that their concentrations are relatively stable and less influenced by the type or proportion of milk used. In contrast, iron content was slightly elevated—though not significantly—in cheese containing 20% donkey milk, potentially reflecting the higher bioavailable iron content in donkey milk. While the differences in trace elements were not statistically significant, the observed trends could have practical relevance, particularly for vulnerable population groups.

### 3.3. Sensory Evaluation of Rolled Cheeses

The acceptability of rolled cheeses was determined by the judgement of 15 women and 9 men, aged 20 to 60 years. Of the 24 assessors, only 8 (33.33%) had previously tasted donkey milk, and only 2 (8.33%) had tasted cheese made from donkey milk.

The sensory attributes of rolled cheeses are reported in Table 5. The color, texture, and overall liking of all rolled cheese samples were above four points (4 = like slightly), while the aroma and taste of rolled cheese made with only cow’s milk were scored between three (3 = neither like nor dislike) and four points (4 = like slightly). A similar observation was made for the taste of the rolled cheese with the addition of 10% donkey’s milk.

A slight increasing trend, though not statistically significant, was observed with the addition of donkey milk in color (*p* = 0.378), aroma (*p* = 0.209), taste (*p* = 0.087), and overall liking (*p* = 0.268).

In our study, the addition of donkey’s milk did not significantly (*p* > 0.05) improve the sensory properties of rolled cheeses. Contrary, a previous study [25] showed that the addition of 5% donkey’s milk in the production of Italian Caciotta cheese improved acceptance parameters after 45 days of ripening. Additionally, the addition of donkey milk (5% and 10%) improved the acceptability of Caprino cheese, an Italian goat cheese [94].

The highest increasing trend was observed in taste, where the rolled cheese with cow’s milk scored 3.50 ± 1.14, while the rolled cheese with the addition of 20% donkey’s milk scored 4.21 ± 0.93. This could be related to the high lactose concentration in donkey milk (6.33%) [74] compared with cow milk (4.6%) [95]. The taste of rolled cheese made from cow’s milk was described as pleasant, milky-sour, and not too salty [96], while cheese made with goat and donkey milk tasted sweet [20].

The triangle test aimed to differentiate between rolled cheeses with the addition of donkey’s milk (10% and 20%) and rolled cheeses made only with cow’s milk. Seventeen (70.83%) assessors identified the different sample when the reference sample (rolled cheese made with 100% cow’s milk) was compared with the rolled cheese with the addition of 10% donkey’s milk, while 19 (79.17%) assessors made the correct judgment when rolled cheese with the addition of 20% donkey’s milk was tested against the reference rolled cheese (rolled cheese made with 100% cow’s milk). The triangle test was significant with *p* < 0.001, considering that a minimum number of 16 assessors was required to establish significance (*p* < 0.001, n = 24).

Although the hedonic scale did not show a significant difference between rolled cheese samples, the triangle test indicated that the addition of donkey’s milk in the process of cheese production has an impact on its overall sensory properties, as previously reported [20,25,94]. Therefore, rolled cheese with the addition of donkey’s milk could find a place in the market.

## 4. Conclusions

Based on the study’s results, the addition of donkey’s milk from the Balkan and Banat breeds in the production of rolled cheese did not influence the microbiological status or sensory attributes of the cheese, but did partially influence its chemical composition and mineral profile. In addition, the differentiation between rolled cheese made from cow’s milk and rolled cheese made with the addition of donkey’s milk, as demonstrated by the triangle test, could encourage the production of innovative and value-added products. However, further research is needed for a true understanding of the effects of donkey milk in the production of rolled cheese with higher inclusion levels of donkey milk or in the production of cheeses with extended ripening times.

## Figures and Tables

**Table 1:** Composition (%), pH, essential minerals, and trace elements in cow’s and donkey’s milk (means ± S.D.).

Composition (Unit)	Cow’s Milk	Donkey’s Milk
Moisture (%)	87.2 ± 0.15	91.4 ± 0.42
Fat (%)	3.93 ± 0.32	0.45 ± 0.29
Non-fat dry matter (%)	8.84 ± 0.18	8.16 ± 0.44
Protein (%)	3.42 ± 0.07	1.67 ± 0.20
Ash (%)	0.61 ± 0.01	0.38 ± 0.05
pH	6.64 ± 0.02	7.23 ± 0.14
Ca ^1^ (mg/kg)	1037 ± 22.5	679 ± 115
P ^2^ (mg/kg)	933 ± 10.0	426 ± 104
Na ^3^ (mg/kg)	572 ± 67.5	379 ± 108
K ^4^ (mg/kg)	836 ± 181	562 ± 173
Mg ^5^ (mg/kg)	144 ± 10.3	79.4 ± 24.1
Zn ^6^ (mg/kg)	3.01 ± 0.29	2.19 ± 0.59
Cu ^7^ (mg/kg)	0.43 ± 0.05	0.42 ± 0.20
Fe ^8^ (mg/kg)	0.37 ± 0.03	0.22 ± 0.05

Notes: ^1^ Ca—calcium; ^2^ P—phosphorus; ^3^ Na—sodium; ^4^ K—potassium; ^5^ Mg—magnesium; ^6^ Zn—zinc; ^7^ Cu—copper; ^8^ Fe—iron.

**Table 2:** Microbiological status of different rolled cheeses (means ± S.D.).

Parameter	Rolled Cheese with Cow’s Milk	Rolled Cheese with the Addition of 10% Donkey’s Milk	Rolled Cheese with the Addition of 20% Donkey’s Milk	*p*-Value	F
Total mesophilic bacteria (log_10_ CFU/g)	5.62 ± 0.26	5.55 ± 0.26	5.68 ± 0.30	0.717	0.34
*Enterobacteriaceae* (log_10_ CFU/g)	2.43 ± 0.33	2.37 ± 0.34	2.67 ± 0.34	0.291	1.34
*Escherichia coli* (log_10_ CFU/g)	1.86 ± 0.28	2.09 ± 0.30	2.29 ± 0.45	0.140	2.25
Coagulase positive staphylococci (log_10_ CFU/g)	<1.00	<1.00	<1.00	-	-
*Salmonella* spp. (25 g)	Not detected	Not detected	Not detected	-	-
*Listeria monocytogenes* (25 g)	Not detected	Not detected	Not detected	-	-

**Table 3:** Composition (%) and pH of different rolled cheeses (Means ± S.D.).

Composition (Unit)	Rolled Cheese with Cow’s Milk	Rolled Cheese with the Addition of 10% Donkey’s Milk	Rolled Cheese with the Addition of 20% Donkey’s Milk	*p*-Value	F
Moisture (%)	49.38 ± 0.66	47.85 ± 1.18	50.44 ± 0.66	0.125	4.50
Fat (%)	24.50 ± 0.00	21.75 ± 0.35	19.75 ± 0.35	0.001	136
Fat in dry matter (%)	48.40 ± 0.63	41.71 ± 0.27	39.85 ± 1.24	0.004	60.3
Protein (%)	20.95 ± 0.16	21.28 ± 1.65	23.19 ± 0.30	0.186	3.11
Protein in dry matter (%)	41.38 ± 0.86	40.84 ± 4.08	46.79 ± 1.23	0.168	3.44
Ash (%)	2.01 ± 0.09	3.26 ± 0.01	2.75 ± 0.07	0.001	175
pH	5.31 ± 0.13	5.51 ± 0.18	5.43 ± 0.08	0.448	1.06
Salt (%)	0.53 ± 0.03	1.93 ± 0.03	1.42 ± 0.03	<0.001	918

**Table 4:** Essential minerals and trace elements (mg/kg) in rolled cheeses (means ± S.D.).

Element (Unit)	Rolled Cheese with Cow’s Milk	Rolled Cheese with the Addition of 10% Donkey’s Milk	Rolled Cheese with the Addition of 20% Donkey’s Milk	*p*-Value	F
Ca ^1^ (mg/kg)	3922 ± 183	3448 ± 0.01	3319 ± 61.8	0.025	16.3
P ^2^ (mg/kg)	3653 ± 178	3397 ± 21.2	3509 ± 4.24	0.187	3.09
Na ^3^ (mg/kg)	2105 ± 134	7600 ± 127	5570 ± 127	<0.001	918
K ^4^ (mg/kg)	380 ± 62.3	986 ± 88.4	953 ± 47.4	0.005	49.9
Mg ^5^ (mg/kg)	115 ± 11.3	95.5 ± 4.95	115 ± 2.12	0.118	4.72
Zn ^6^ (mg/kg)	27.4 ± 2.97	27.9 ± 0.14	31.4 ± 0.28	0.181	3.19
Cu ^7^ (mg/kg)	8.00 ± 0.57	9.95 ± 0.07	11.8 ± 3.04	0.246	2.32
Fe ^8^ (mg/kg)	9.10 ± 1.13	9.90 ± 2.55	13.4 ± 5.44	0.520	0.82
Ca/P ratio	1.07 ± 0.01	1.02 ± 0.01	0.95 ± 0.02	0.003	79.8

Notes: ^1^ Ca—calcium; ^2^ P—phosphorus; ^3^ Na—sodium; ^4^ K—potassium; ^5^ Mg—magnesium; ^6^ Zn—zinc; ^7^ Cu—copper; ^8^ Fe—iron.

**Table 5:** Consumer acceptability of rolled cheeses (means ± S.D.).

Parameter	Rolled Cheese with Cow’s Milk	Rolled Cheese with the Addition of 10% Donkey’s Milk	Rolled Cheese with the Addition of 20% Donkey’s Milk	*p*-Value	F
Color	4.04 ± 0.95	4.25 ± 0.99	4.42 ± 0.83	0.378	0.99
Texture	4.00 ± 0.88	4.33 ± 0.82	4.13 ± 1.03	0.449	0.81
Aroma	3.79 ± 0.93	4.21 ± 1.02	4.21 ± 0.83	0.209	1.60
Taste	3.50 ± 1.14	3.75 ± 1.22	4.21 ± 0.93	0.087	2.53
Overall liking	4.00 ± 1.02	4.13 ± 0.90	4.42 ± 0.78	0.268	1.34

## Data Availability

The original contributions presented in the study are included in the article, further inquiries can be directed to the corresponding author.
